# Intraoperative oxygen tension and redox homeostasis in Pseudomyxoma peritonei: A short case series

**DOI:** 10.3389/fonc.2023.1076500

**Published:** 2023-01-26

**Authors:** Francisca Valenzuela-Molina, Florina I. Bura, Mari C. Vázquez-Borrego, Melissa Granados-Rodríguez, Blanca Rufián-Andujar, Sebastián Rufián-Peña, Ángela Casado-Adam, Juan Manuel Sánchez-Hidalgo, Lidia Rodríguez-Ortiz, Rosa Ortega-Salas, Ana Martínez-López, Carmen Michán, José Alhama, Álvaro Arjona-Sánchez, Antonio Romero-Ruiz

**Affiliations:** ^1^ Surgical Oncology Unit, Department of Surgery, Reina Sofia University Hospital, Cordoba, Spain; ^2^ GE09 Research in peritoneal and retroperitoneal oncological surgery, Maimonides Biomedical Research Institute of Cordoba (IMIBIC), Reina Sofia University Hospital, University of Cordoba, Cordoba, Spain; ^3^ Pathology Unit, Reina Sofia University Hospital, Cordoba, Spain

**Keywords:** Pseudomyxoma peritonei, mucin, oxygen, hypoxia, HIF-1α, catalase, ROS

## Abstract

**Introduction:**

Pseudomyxoma peritonei (PMP) is a rare malignant disease characterized by a massive multifocal accumulation of mucin within the peritoneal cavity. The current treatment option is based on complete cytoreductive surgery combined with hyperthermic intraperitoneal chemotherapy. However, the recurrence is frequent with subsequent progression and death. To date, most of the studies published in PMP are related to histological and genomic analyses. Thus, the need for further studies unveiling the underlying PMP molecular mechanisms is urgent. In this regard, hypoxia and oxidative stress have been extensively related to tumoral pathologies, although their contribution to PMP has not been elucidated.

**Methods:**

In this manuscript, we have evaluated, for the first time, the intratumoral real-time oxygen microtension (pO2mt) in the tumor (soft and hard mucin) and surrounding healthy tissue from five PMP patients during surgery. In addition, we measured hypoxia (Hypoxia Inducible Factor-1a; HIF-1α) and oxidative stress (catalase; CAT) markers in soft and hard mucin from the same five PMP patient samples and in five control samples.

**Results:**

The results showed low intratumoral oxygen levels, which were associated with increased HIF-1α protein levels, suggesting the presence of a hypoxic environment in these tumors. We also found a significant reduction in CAT activity levels in soft and hard mucin compared with healthy tissue samples.

**Discussion:**

In conclusion, our study provides the first evidence of low intratumoral oxygen levels in PMP patients associated with hypoxia and oxidative stress markers. However, further investigation is required to understand the potential role of oxidative stress in PMP in order to find new therapeutic strategies.

## 1 Introduction

Pseudomyxoma peritonei (PMP), also known as “Jelly belly”, is a rare condition characterized by the progressive and abundant accumulation of mucinous ascites and peritoneal implants (1–3). The most prevalent cause of this disease is a perforated epithelial tumor of the appendix (4). In the early stages of the disease, the absence of symptoms promotes the accumulation of large volumes of mucin inside the abdomen, eventually leading to severe abdominal enlargement, pain, and malnutrition (5, 6). Intestinal obstruction occurs over time as a consequence of compression on visceral organs, triggering an inflammatory and fibrotic mesothelium response that can be fatal in patients with untreated or recurrent PMP (4–6).

The Peritoneal Surface Oncology Group International (PSOGI) classification, which has been validated on several occasions (7, 8), divides PMP into four groups based on histological characteristics: i) acellular mucin (AM), ii) low-grade mucinous carcinoma (LG-PMP), iii) high-grade mucinous carcinoma (HG-PMP), and iv) PMP with the presence of signet ring cells (SRC) (4). However, histopathology does not completely predict tumor behavior (9). On the other hand, mucin from PMP is classified into soft, semi-hard, and hard mucin samples based on physical and chemical properties as well as visual appearance (10).

Under normal cellular conditions, mitochondrial respiration and NADPH oxidases generate reactive oxygen species (ROS), which are scavenged by cellular non-enzymatic and enzymatic antioxidant mechanisms such as superoxide dismutase [cytosolic (SOD1) and mitochondrial (SOD2)] and catalase (CAT), among others (11, 12). The imbalance between ROS production and their elimination causes oxidative stress, which has been associated with pathogenetic mechanisms in various diseases, including cancer (12, 13). In this sense, ROS overproduction can lead to cellular transformation by activating a wide range of signaling factors such as extracellular signal-regulated kinase 1/2 (ERK1/2), receptor tyrosine kinases (RTK), vascular endothelial growth factor (VEGF) and hypoxia inducible factor-1α (HIF-1α) to promote cellular proliferation, invasion, metastasis, and angiogenesis (14, 15). In addition to ROS, tumoral hypoxia is also responsible for HIF-1α upregulation, which in turn regulates the expression of proangiogenic, antioxidant, and goblet cell-associated factors, as well as mucin genes, allowing cancer cells to survive, proliferate, and undergo epithelial to mesenchymal transition (EMT) (16–18). However, the potential role of hypoxia and/or oxidative stress in PMP remains unknown.

This study aims to demonstrate, both *in vitro* and *in vivo*, that PMP is a tumor that grows in the absence of oxygen. Thus, we used intraoperative micro-oximeters, which had previously been used in other surgical disciplines (19–22) but had never been used for this purpose, to make intratumoral measurements of the real-time oxygen levels during the surgical procedure in PMP patients. Furthermore, we correlated the intraoperative pressure of oxygen values with measured cellular hypoxia and oxidative stress markers in tissue samples from these patients.

## 2 Material and methods

### 2.1 Patients and samples

This is a prospective analytic study performed in patients with PMP who underwent cytoreductive surgery combined with hyperthermic intraperitoneal chemotherapy (CRS + HIPEC) in our unit. The present study was included in the PI19/01603 study entitled “Molecular characterization of Pseudomyxoma peritonei and development of new therapeutic targets and biomarkers in a PMP xenograft human model˝, funded by Carlos III Research Institute in 2019. The Cordoba Research Ethics Committee reviewed and generated a favorable dictamen for this study on May 28^th^, 2019. All the patients were informed and signed the informed consent form.

We determined the real-time oxygen microtension (pO_2_mt) in tumoral and surrounding healthy tissues in five PMP (four from appendiceal origin and one from caecum-appendiceal stump origin) patients during the CRS + HIPEC (**Table 1**). Furthermore, hypoxia and oxidative stress markers in soft and hard mucin from the same 5 patient samples and in 5 different control samples were evaluated (3 appendix samples from a prophylactic appendectomy due to another medical condition and 2 normal colon samples from PMP patients). Until processing, all samples were stored at -80°C. Moreover, all samples were histologically studied by experienced pathologists to confirm the diagnosis.

**Table 1:** Descriptive characteristics of the study population.

*Variable*	*Descriptive Results*
*Sex (F/M)*	4 (80%)/1(20%)
*Age (years)*	64 (57-79)
*Peritoneal Cancer Index*	27 (22-31)
*Histological grade*	• LG= Patient 3 and 5 (40%) • HG= Patient 1and 2 (40%) • SRC= Patient 4 (20%)
*FiO_2_ (%)*	50 (50-58)
*SaO_2_ (%)*	99.4 (96-100)
*Temperature (°C)*	37 (36.8-37.2)

FiO2: Fraction of inspired oxygen; SaO2: Arterial Oxygen Saturation; LG; low grade; HG: high grade; and SRC: high grade-signet ring cells. The percentage of patients in each category or the value range for the different parameters are indicated in brackets. The median and interquartile range is described for continue variables.

OxyLite™ monitors (Oxford Optronix Ltd. United Kingdom) and specific probes were used to assess the pO_2_mt of tumor tissue (soft and hard mucin) following the manufacturer´s instructions. This oxygen monitors use a fluorescence-based technique to provide an absolute measurement of dissolved oxygen in mmHg or kPa, giving a direct readout of the balance between oxygen supply and oxygen consumption. The oxygen sensors are based on fluorescence quenching and fiber-optic technology. To excite a platinum-based fluorophore linked to the sensor tip, short pulses of LED light are transmitted along the fiber optic sensor. The instrument detects the resulting emission of fluorescent light, which is quenched by the presence of oxygen molecules. The instrument measures fluorescence lifetime, which is inversely proportional to dissolved oxygen levels and can be used to calculate absolute oxygen values in mmHg or kPa. The oxygen microsensors are all made of optical fibers with an outer diameter of 230μm. The sensors used have a 350μm tip diameter and a minimally invasive ‘bare-fiber’ format (with integrated temperature sensor). Temperatures variations have a minimum effect on fluorescence-based oxygen detection. To achieve the highest level of precision, we used sensors with integrated temperature detectors and monitors with constant temperature input (provided by the thermocouple).

Before performing any intratumor oxygen measurements and after the anesthetic induction, each patient´s haemoglobin oxygen saturation (SaO_2_), body temperature, and ventilator-determined inspired fraction of oxygen (FiO_2_) were checked. Moreover, arterial blood gasometry was extracted to obtain the oxygen pressure (mmHg) values in the blood at the moment of micro-oxygenation measurement. To acquire the pO_2_mt for each fraction, micro-oxygenation probes were placed into i) soft free mucin lakes in each of the abdominal quadrants, ii) hard mucin identified by regions, and iii) healthy tissue, for instance, colon or abdominal wall. The probe was placed gently inserted, trying to cover the tip of the fiber sensor with surrounding tissue. We waited at least 15 seconds for a consistent value to be seen before deciding on it as the pO_2_mt of that area. The procedure method is supported by video media material (**Supplemental videos 1-5**).

### 2.2 Sample processing for molecular determinations

Soft mucin samples (~2mL) were homogenized in 4mL of lysis buffer [50mM Tris HCl pH 7.5, 1mM thylenediaminetetraacetic acid (EDTA) and 1mM phenylmethylsulfonyl fluoride (PMSF)] and control and hard mucin samples (~2g) were homogenized in 8mL of lysis buffer, using Potter homogenizers kept on ice in all cases. After homogenization, samples were vacuum filtered with Whatman filter paper before proceeding to a centrifugation phase (16000g, 15min, 4°C). Finally, supernatants were collected, aliquoted and stored at -20°C until assays were performed.

### 2.3 Catalase enzymatic assay

A 20mM KH_2_PO_4_ buffer (pH 7) and a 1M solution of H_2_O_2_ in KH_2_PO_4_ buffer were used to determine catalase activity (23). A water bath set at 30°C with circulating water, coupled to a spectrophotometer set at 240nm, was also required. For each sample, 970μL of KH_2_PO_4_ buffer, 20μL of 1M H_2_O_2_ solution and 10μL of supernatant obtained after sample processing, were added to a cuvette and mixed by inversion. The protocol established for this assay was to measure the absorbance (in triplicate) every 5s for 60s, yielding a unique value of ΔAbs/min per sample. The obtained values were used to calculate total enzymatic activity. Then, those results were extrapolated to get the specific catalase activity for each sample taking into account the protein quantification.

### 2.4 Western blot

The protein concentration of the samples was determined using the Bradford assay (Bio-Rad) (24). Then, 25μg of total protein was subjected to SDS-PAGE on 12% polyacrylamide gels, electro-transferred on polyvinylidene difluoride membranes (Millipore) using the Trans-Blot Turbo system (Bio-Rad), and incubated overnight at 4°C with the corresponding primary antibody [anti-GAPDH (1/100000; Abcam) and anti-HIF-1α (1/500; Cell Signaling)]. After primary antibody incubation, membranes were incubated with horseradish peroxidase-conjugated secondary antibody [anti-rabbit (1/5000; Abcam)]. For protein detection, chemiluminescence ECL Western Blotting Substrate (Thermo Scientific) was used. Protein levels were normalized using TPN (Total Protein Normalization) method, which calculates the intensity value of total protein from each sample. Densitometric analysis of protein bands was conducted using ImageJ.

### 2.5 Statistics

Statistical analyses were performed using Prism software v.7.0 (GraphPad Software, La Jolla, CA, USA). Data were assessed for normality using Kolmogorov-Smirnov test, then evaluated using appropriate parametric (unpaired t test or one-way ANOVA followed by *post hoc* Tukey test) or nonparametric (Mann-Whitney test) tests. *P*-values less than 0.05 were considered significant. Asterisks (* *p*< 0.05, ** *p*< 0.01, *** *p*< 0.001) indicate statistically significant differences.

## 3 Results

### 3.1 Intraoperative oxygen pressure in PMP

**Figure 1:**
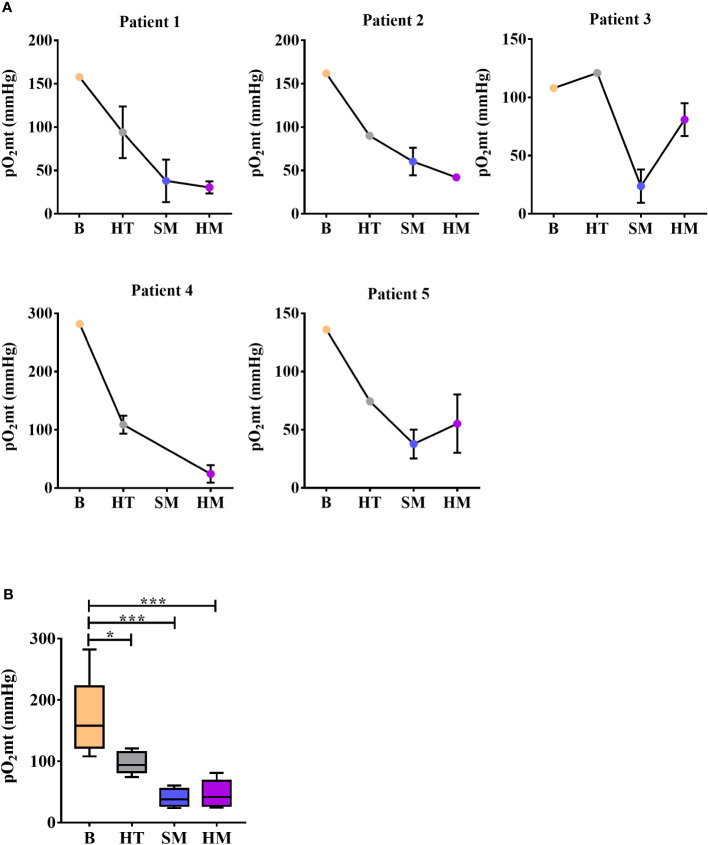
Measurement of intraoperative real-time oxygen microtension (pO_2_mt) in blood and tissues during surgery. **(A)** Individual measurements of pO_2_mt (mmHg) in blood (B), surrounding healthy tissue (HT), soft mucin (SM) and hard mucin (HM) in each PMP patient (n=5). Measurements are represented as the mean ± S.E.M of all values measured per zone in each patient. **(B)** pO_2_mt (mmHg) levels per zone taking into account all patients included in the study (n=5). One-way ANOVA analysis was carried out with multiple comparisons. *p < 0.05, ***p < 0.001.

The pO_2_mt was measured intratumorally in soft free mucin, and hard mucin from all the patients included in the study, as well as in blood and surrounding healthy tissues. Interestingly, the pO_2_mt was clearly reduced in soft and hard mucin tissue compared with blood and healthy tissue in all patients. In patient 4, it was not possible to measure the pO_2_mt in soft mucin since the tumor was mainly constituted by hard mucin (**Figure 1A**). Additionally, the statistical analysis of all patients showed significant differences between soft and hard mucin compared with blood and healthy tissue (**Figure 1B**). Moreover, the O_2_ levels were maintained under normal parameters in all the patients as shown by FiO_2_ and SaO_2_ parameters (**Table 1**).

### 3.2 Determination of hypoxia and oxidative stress markers

**Figure 2:**
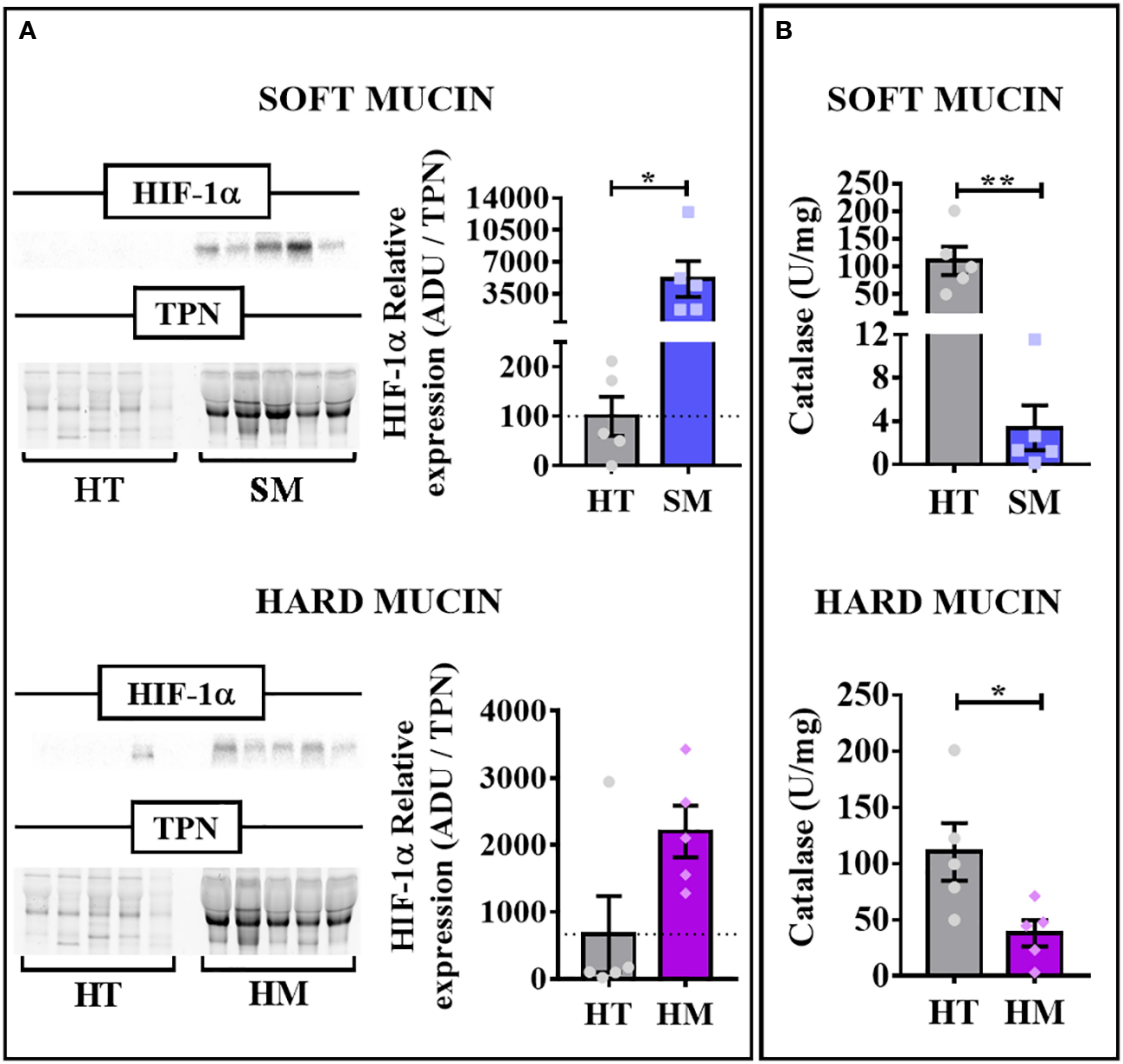
Cellular hypoxia and oxidative stress markers in PMP samples. **(A)** HIF-1α relative protein expression levels in soft (SM; n=5) and hard mucin (HM; n=5) compared with healthy tissues (HT; n=5; non-tumoral appendix and colon tissues) evaluated by Western Blot. The arbitrary densitometric unit (ADU) for the protein was normalized by the Total Protein Normalization (TPN) value. **(B)** Catalase activity (U/mg) in soft and hard mucin (n=5) compared to healthy control tissues (n=5; non-tumoral appendix and colon tissues) evaluated by enzymatic assay. Unpaired t test or Mann-Whitney test was used based on Kolmogorov-Smirnov normality test. *p < 0.05, **p < 0.01.

Next, we measured hypoxia and oxidative stress markers in order to know if PMP develops not only due to the absence of oxygen but also due to an increase in ROS. In this sense, we found a clear increase in HIF-1α protein expression levels in soft and hard mucin compared with healthy tissue samples (**Figure 2A**), which supports the reduction of pO_2_mt observed in the patients. Additionally, we evaluated the catalase enzymatic activity as an oxidative stress marker in our patient´s samples. In this case, and in contrast with the HIF-1α results, we observed a significant reduction of catalase activity in soft and hard mucin compared with healthy tissue samples (**Figure 2B**).

## 4 Discussion

The scarce research in PMP is mainly focused on the clinical management and classification of the disease, and the small number of articles exploring the PMP pathophysiology are based on histological data and expression analyses of genes already described in other types of cancer (25–27). In this context, it has been postulated that mucinous tumors, like PMP, might use the mucin barrier to configure a favorable local environment for tumor growth (28). In this study, we have demonstrated for the first time that the tumor tissue in PMP grows in hypoxic conditions increasing hypoxia markers such as HIF-1α. This finding is consistent with the fact that most solid malignant diseases show low oxygen levels (hypoxia) in the tumor environment (29–31), increasing the resistance to chemotherapy and radiotherapy.

Cellular adaptation to hypoxia is triggered by overexpression of HIF-1α *via* modulation of different signaling pathways to increase blood vessels formation, aggressiveness, metastasis, and resistance to treatments in numerous cancer types, such as breast, colorectal and pancreas cancer (17, 29, 31–35). Here, we measured HIF-1α protein levels to confirm the low levels of oxygen detected *in vivo* in PMP patients. We found a significant increase in HIF-1α protein levels in soft and hard mucin samples compared with healthy tissue samples. In line with these findings, Dilly et al. have shown an increase in HIF-1α protein levels in PMP tissues compared with normal colon samples, as well as an increase in HIF-1α levels in LS174T colon cancer cell line and PMP tissue explants following exposure to hypoxic conditions (18).

Moreover, HIF-1α has been reported to be modulated by hypoxia-independent factors, including ROS (29, 36). In this sense, we evaluated the CAT enzymatic activity as an oxidative stress marker in order to know if the hypoxic environment found in PMP is also linked to ROS. Thus, we have described a significant reduction in CAT enzymatic activity in soft and hard mucin compared with healthy tissue samples. Interestingly, CAT has been described as downregulated in several tumoral pathologies (37–41) while, at the same time, being elevated in other cancer subtypes (38, 42, 43). This discrepancy is due to its complex regulatory process. In this regard, CAT has been reported to be regulated at different levels: transcriptional (high number of transcription factors that induce or repress the transcriptional activity of CAT promoters), post-transcriptional (mRNA stability), and post-translational (phosphorylation and ubiquitination). Furthermore, epigenetic or genetic changes may have a role in regulating CAT activity (44).

Due to the harsh environment developed in mucinous tumors, which is characterized by hypoxic conditions, low pH, and no vascularization (45), conventional chemotherapy (e.g., bleomycin or doxorubicin, both oxygen-dependent drugs) and radiotherapy are ineffective in most cases (46–48). As a result, several research groups have concentrated on seeking suitable therapeutic options for oncologic patients suffering from this type of tumor by different means. Thus, Dilly et al. showed a reduction in MUC2 expression levels using a HIF-1α siRNA or specific HIF-1α inhibitors (BAY 87-2243 and YC-1) in LS174T cell line. Interestingly, chronic BAY 87-2243 treatment also reduced mucinous tumor growth in a PMP xenograft mouse model (18). Indeed, modulation of the HIF pathway at different levels has been proposed as a promising cancer therapy (49). Thus, transcription and translation have been demonstrated to be suppressed by different compounds, including aminoflavone, anthracyclines, steroids, or topoisomerase inhibitors. Remarkably, digoxigenin inhibited HIF-1α translation, increasing pancreatic cancer cells’ sensitivity to gemcitabine (49). Moreover, several histone deacetylase (HDAC) inhibitors, such as Panobinostat, MPT0G157, and Vorinostat, have been reported to reduce HIF-1α stability and induce its degradation in different tumoral pathologies (49). In line with this, Kim et al. reported the use of trichostatin A, a HDAC inhibitor, to downregulate HIF-1 and hypoxia-induced angiogenesis in an *in vivo* Lewis lung carcinoma model (50). Additionally, Kishimoto et al. proposed using hypoxia-activated prodrugs such as Evofosfamide in combination with radiotherapy and antiproliferative treatments to improve the clinical outcome of cancer patients (48).

On the other hand, harnessing ROS-induced oxidative stress *via* targeted inhibition of the cancer antioxidant defense machinery has been proposed as a potential anticancer strategy (51). In this sense, ATN-224 (choline tetrathiomolybdate) is an orally bioavailable inhibitor that inhibits SOD activity *via* copper chelation. This inhibitor has been used in phase I and II clinical trials and has been related to reduced angiogenesis and tumor proliferation (52, 53). In the same line, 4,5-dichloro-2-(3-tolyl)pyridazin3(2H)-one (LSC-1) has been related to the reduction of lung adenocarcinoma cell growth through SOD inhibition (54, 55). Furthermore, the combination of Capecitabine (a prodrug that is converted to 5-FU by thymidine phosphorylase) and Celecoxib (a Cox-2 inhibitor) reduced the number of mice with pancreatic adenocarcinoma, which was associated with a recovery of SOD and CAT activity (56).

In conclusion, this work provides the first evidence of low intratumoral oxygen levels in PMP patients during surgery, as well as an increase in HIF-1α protein levels together with low catalase activity, suggesting the presence of a hypoxic environment in these tumors. Further research is required to deeply understand the mechanisms underlying hypoxia and the potential role of oxidative stress in this pathology in order to identify new targets and strategies to treat this devastating disease.

## Data availability statement

The original contributions presented in the study are included in the article/**Supplementary Material**. Further inquiries can be directed to the corresponding authors.

## Ethics statement

The studies involving human participants including the use of OxyLiteTM systems were reviewed and approved by Cordoba Research Ethics Committee (protocol code PI19/01603). Written informed consent to participate in this study was provided by all the patients/participants. 

## Author contributions

Conception and design: AA-S, MV-B, AR-R; Development of methodology: AA-S, MV-B, AR-R, FV-M, FB, MG, CM, JA; Acquisition of samples and data: FV-M, BR-A, SR-P, AC-A, JS-H, LR-O; Analysis and interpretation of data: AA-S, MV-B, AR-R, FV-M, FB, CM, JA. All authors contributed to the article and approved the submitted version.
